# Papillary renal neoplasm with reverse polarity with a favorable prognosis: Two cases report and literature review

**DOI:** 10.3389/fonc.2022.1011422

**Published:** 2022-10-31

**Authors:** Zuo Yuzhi, Liang Zhen, Xiao Yu, Pan Boju, Yan Weigang, Wu Xingcheng

**Affiliations:** ^1^ Department of Urology, Peking Union Medical College Hospital, Peking Union Medical College, Chinese Academy of Medical Sciences, Beijing, China; ^2^ Department of Pathology, Peking Union Medical College Hospital, Peking Union Medical College, Chinese Academy of Medical Sciences, Beijing, China

**Keywords:** renal cell carcinoma, reverse polarity, papillary renal cell carcinoma, pathology, partial nephrectomy

## Abstract

**Background:**

Papillary Renal Neoplasm (PRN) with polarity inversion is a less common subtype of kidney cancer with an apparently recognizable morphology, distinct immunohistochemical profiles, and frequent *KRAS* mutations. It has been estimated to account 4% of previously diagnosed PRN.

**Case presentation:**

This is a retrospective case report of two patients diagnosed with PRNRP. Two males were found to have kidney mass accidentally through imaging examination in clinic. Both of the patients had no obvious discomfort and abnormal test indicators. Subsequently, they underwent partial nephrectomy in our center by the same surgeon and followed up closely with an impressive clinical outcome. The pathology reports indicated that their pathological features were consistent with PRNRP. The HE staining showed a monolayer of papillary or tubular structures, with small nuclei away from the cytoplasmic top of the basement membrane. The immunohistochemical results were GATA3 (+), vimentin (-).

**Conclusion:**

Our case reports and literature review suggested that PRNRP should be separated from traditional PRN and partial nephrectomy is a robust modality for PRNRP. The morphological, immunohistochemical, and genetic information of the cases we presented would provide important material for PRNRP to become a distinct category with benign clinical outcome.

## Introduction

Papillary renal neoplasm (PRN) is the second most common subtype of kidney cancer, which takes up 15%-20% of renal cell carcinoma (RCC) (1, 2). PRN with reverse polarity (PRNRP) is a rare and newly defined subtype of PRN, accounting for 4-8.6% of the total (3–5). In 2019, Al-Obaidy et al. named the diagnosis for the first time as PRNRP and demonstrated that it was different from common PRN in histomorphological, immunohistochemical, and chromosomal features (3). This tumor type has been described as being characterized by branching papillae, or rarely, predominant tubules covered by bland oncocytic cells with apical low- International Society of Urological Pathology (ISUP)-grade nuclei. This article would report two cases PRNRP and review the current literature reports. We believe our results would offer new insights into PRNRP, thus aiding future efforts for the diagnosis and treatment of this disease. Both patients have fulfilled the written informed consent.

## Case presentation

### Case 1

**Figure 1:**
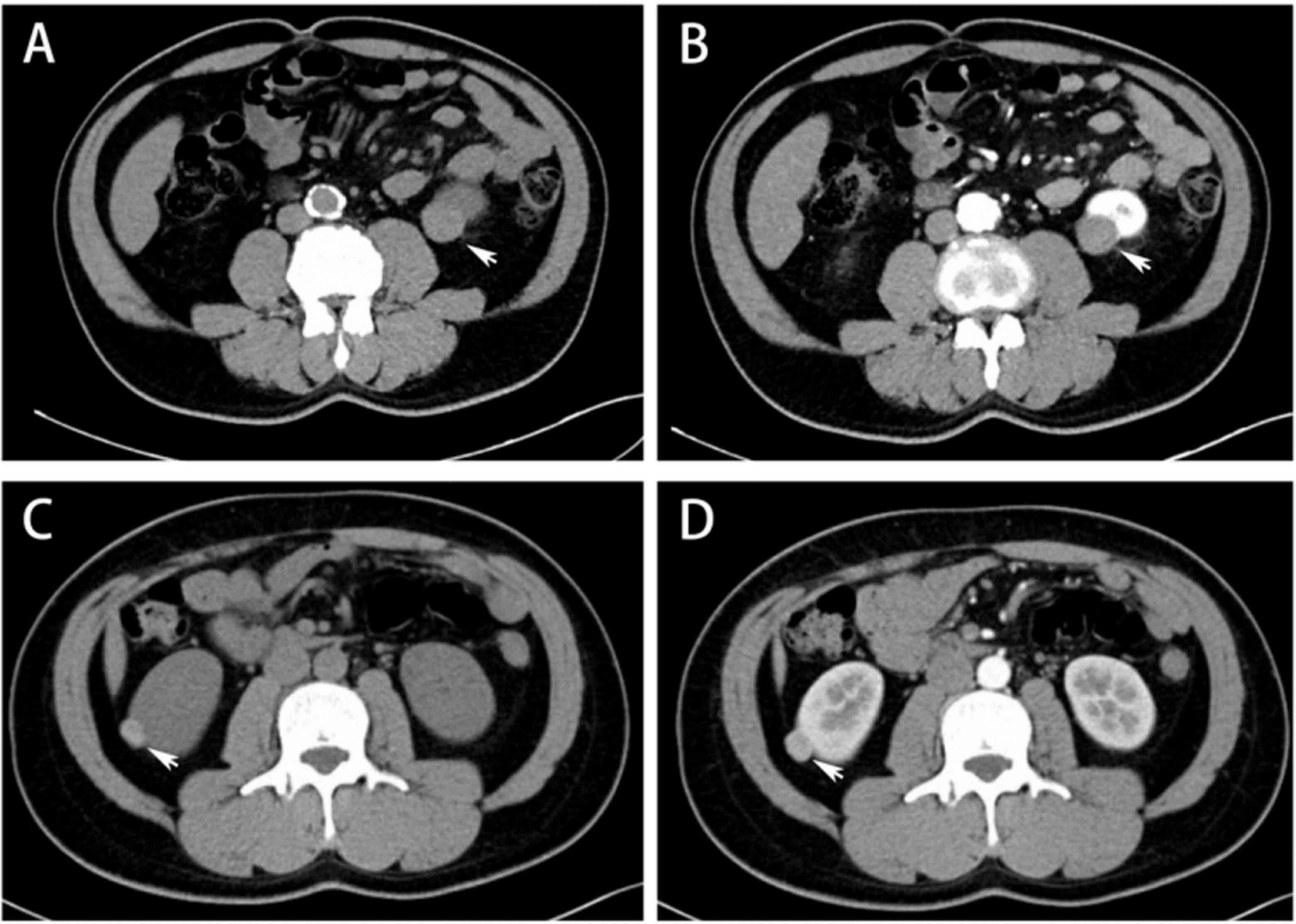
**(A, B)** Computed tomography revealed a slightly high-density nodule at the lower pole of the left kidney for case one, with a diameter of about 2.5 cm and a CT value of 57 HU on plain scan with no obvious strengthening. **(C, D)** CT demonstrated a 1.6×1.3 cm round high-density nodule in the right kidney of case 2 with a CT value of about 80HU on plain scan. No enhancement after intravenous administration of contrast was noted.

The patient is a 54-year-old male, who was admitted to the hospital due to the discovery of a left kidney tumor by ultrasound. The patient had no complains of clinical symptoms, no fever, low back pain, or hematuria. He was a heavy smoker (30 pack/year). Urine culture and urine cytology of that patents demonstrated normal results. The results of blood routine, liver and kidney function, coagulation test indexes also showed no obvious abnormality. Ultrasonography of the urinary system demonstrated mixed echoes in the lower pole of the left kidney, which measured 4×3 cm in size, with a clear boundary, thick septa. A 1.1×1.0 cm regular shape, medium-high echo was detected on the wall. Enhanced CT showed a slightly high-density nodule at the lower pole of the left kidney, with a diameter of about 2.5 cm and a CT value of 57 HU on plain scan with no obvious strengthening (**Figures 1A, B**). Based on Bosniak criteria (6), the lesion was sorted as category IV. Laparoscopic left partial nephrectomy was then performed. The tumor showed a complete capsule, and the section composed of cystic and solid components.

The postoperative pathology revealed PRNRP (grade 2 PRN, stage pT1a). The tumor incision is grayish red with medium texture and fine papillary shape. Tumor was confined to the kidney, with no extension into the perinephric adipose tissue, renal sinus, renal pelvis, or Gerota fascia. Immunohistochemical phenotypes were as follow: GATA3 (+), Keratin 34βe12 (localized +), AE1/AE3 (+), Keratin 7 (+), EMA (+), vimentin (-), PAX-8(-), CD10(-), CD117(-), CA9(-), TFE3(-), RCC(-), P504(-). After one year of follow-up, the patient recovered well without disease progression or recurrence.

### Case two

The patient was a 35-year-old male, who admitted to the hospital due to physical examination of a right kidney mass for 1 week. The patient had no clinical manifestations, no fever, low back pain, or hematuria. The findings of laboratory tests, including complete blood count, liver and renal function tests, and urine analysis, were within normal range. The patient was previously healthy with no family history of tumor.

Ultrasound of the urinary system demonstrated an anechoic, regular mass at the middle and lower part of the right kidney measuring 1.9×1.6×1.3 cm in size, with clear boundary. Enhanced CT showed a 1.6×1.3 cm round high-density nodule in the right kidney with a CT value of about 80HU on plain scan. No enhancement after intravenous administration of contrast was noted. The mass was classified as Bosniak type III (**Figures 1C, D**). Chest CT showed a 4mm nodules at upper lobes.

Laparoscopic right partial nephrectomy was then performed, and the postoperative specimen showed a complete capsule, with solid and cystic changes in cut section. The pathological study confirmed the diagnosis of PRNRP polarity, grade 2 with 20% of necrosis, without vascular embolus or peri-renal infiltration, pT1aNx. No renal capsule invasion, no renal pelvis and renal sinus fat involvement was detected. Immunohistochemical phenotypes: GATA3(+), AE1/AE3(+), Keratin 7(+), EMA(+), vimentin(-), PAX-8(-), CD10(-), CD117(-), CA9(-), TFE3(-), RCC(-), P504(-). The patient did not report any specific discomfort and disease progression at his one year follow-up.

**Figure 2:**
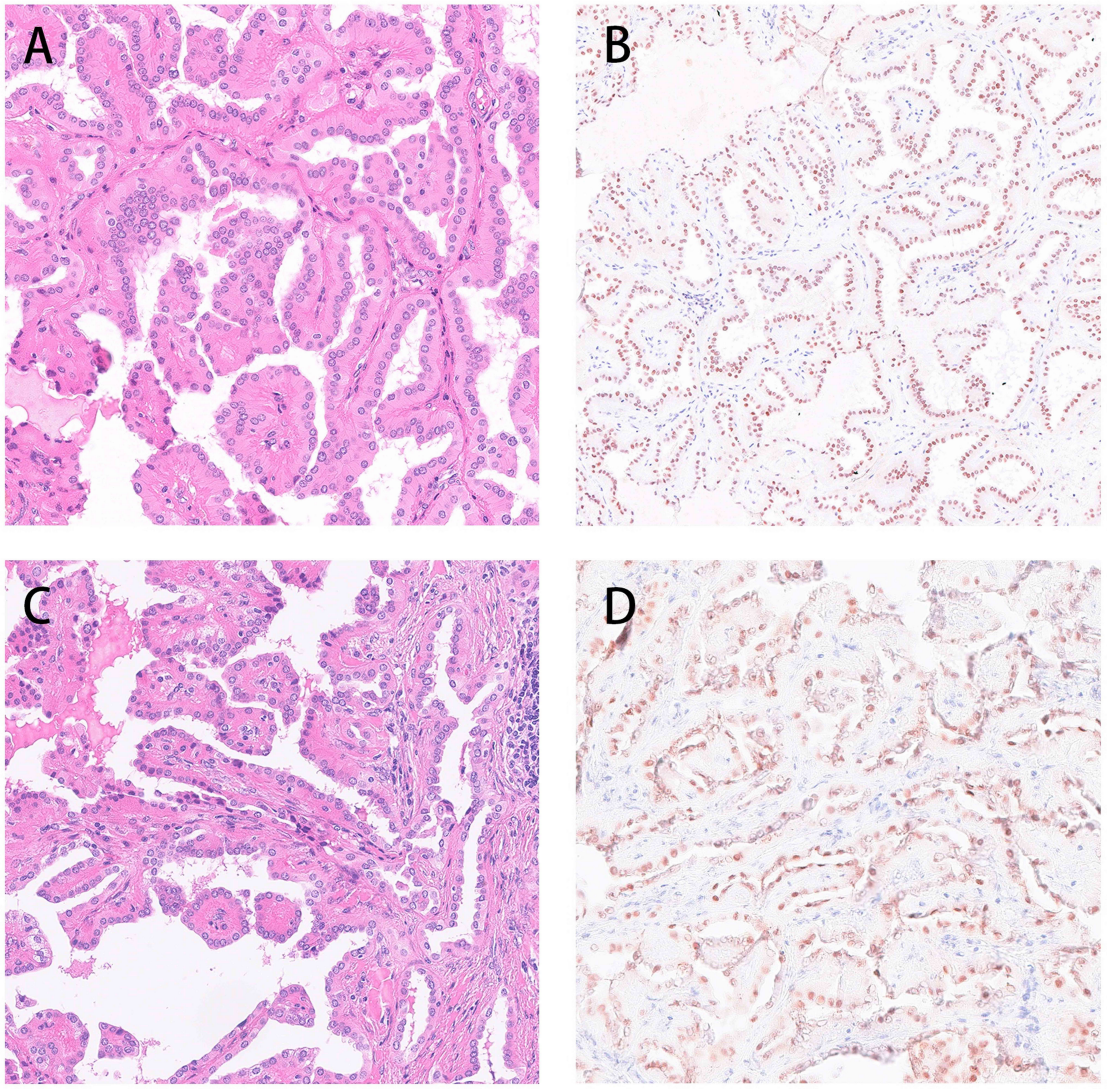
Both the pathological manifestations of the tumors from the aforementioned two patients were consistent with typical PRNRP. The HE staining showed papillary or tubular structures, the surface was covered with a monolayer of eosinophils, the cytoplasm was granular, with small nuclei away from the cytoplasmic top of the basement membrane. The immunohistochemical phenotype was GATA3 (+), vimentin (-) (**A, B**: Case 1 **C, D** Case 2).

Both the pathological manifestations of the tumors from the aforementioned two patients were consistent with typical PRNRP. The HE staining showed papillary or tubular structures, the surface was covered with a monolayer of eosinophils, the cytoplasm was granular, with small nuclei away from the cytoplasmic top of the basement membrane. The immunohistochemical phenotype was GATA3 (+), vimentin (-) (**Figure 2**).

## Discussion

With the development of molecular biology and genetics, PRN including three molecular subtypes with different prognostic propensities based on whether it is combined with *CDKN2A* gene silencing, *SETD2 gene* mutation, *TFE3* gene fusion, or increased expression of *NRF2*–antioxidant response element signaling pathway (7). PRNRP is pathologically characterized as papillary or tubular structures covered with a monolayer of eosinophilic cells with granular cytoplasm and small, inconspicuous nuclei located at the cytoplasm away from the basement membrane (8). Immunohistochemistry showed that GATA3 and L1CAM were positive, but vimentin was negative (9). Fluorescence *in situ* hybridization (FISH) analysis demonstrated a low level of chromosomal abnormalities (10). Trisomy 7/17 was found in 20% of tumors, and chromosome Y deletion was found in 14% of male patients. All the aforementioned pathology characteristics are significantly different from typical PRN (9).

Genetically, 80-100% of PRNRP would activate mutations in* KRAS* (4, 8, 11), which is relatively rare in other types of RCC. According to TCGA database (The Cancer Genome Atlas database), only 0.4% (2/488) of clear cell RCC have *KRAS* gene mutations; about 1.8% (5/274) of PRN have *KRAS* gene mutations; no *KRAS* gene mutation was detected in chromophobe RCC (7, 11, 12). PRNRP is the only pathological subtype of RCC with a high frequency of *KRAS* mutations. *KRAS* gene belongs to *RAS* gene family, which can encode p21 protein with GTPase activity. It forms a classic tyrosine kinase signal pathway with downstream *raf/mek/erk* and *MAPK*, and plays an important role in regulating cell proliferation, differentiation, migration and apoptosis (13, 14). *KRAS* gene is an important proto-oncogene, and its abnormal activation is associated with a variety of malignant tumors, such as lung cancer, colon cancer and pancreatic cancer (15, 16). Different tumor types have different *KRAS* mutation profiles. For example, p.Gly12Cys and p.Gly12Val missense mutations are common in non-small cell lung cancer, whereas p.Gly12Asp missense mutations are more likely to occur in colorectal cancer (17, 18). Different *KRAS* gene mutations can produce different downstream effects, thus affecting the prognosis of the disease. In non-small cell lung cancer, patients with p.Gly12Cys and p.Gly12Val mutations would promote RAS-related protein signaling (v-ral simian leukemia viral oncogene homolog *ras* related, *Ral* signaling) and reduce the activation of growth factor-dependent *AKT* (growth factor-dependent *Akt*) thus leads to a worse prognosis than patients with other mutations; whereas p. Gly12Asp mutation plays critical a role by increasing the activation of *PI3K* (phosphatidylinosi-tol 3-kinase) and *MEK* (mitogen-activated protein/extracellular signal-regulated kinase kinase). In PRNRP, the common *KRAS* gene mutation sites are p.Gly12Val, p. Gly12Asp and p. Gly12Arg, accounting for 61.5%, 34.6% and 3.9%, respectively (11). Nevertheless, the effect of different genotypes on clinical prognosis remains to be unclear.

The mean onset age of PRNRP was around 60 years, with no significant gender differences. Like common RCC, PRNRP may not generally demonstrate any symptoms in the early phase and may be found accidentally through physical examination. A few patients may have symptoms of waist discomfort or hematuria. In present study, The CT imaging features of the two cases were both high-density cysts without obvious enhancement. Tong et al. demonstrated that 5 of 12 of their cases were cystic (19). Chang et al. also demonstrated that 6 of their 10 tumors were cystic (4). The cystic features were also proved by the loose papillae floating in the large spaces shown by the images in the published cases (20). Thus we can infer that CT images features of PRNRP were small-sized, well circumscribed neoplasm, often encapsulated and cystic, with no obvious enhancement. According to previous study, the majority of the patients had tumors less than 3 cm in diameter, TNM staging was mainly T1aN0M0, and the mainstay of treatment is partial nephrectomy. The biological behavior of PRNRP is generally indolent, with promising prognosis. Up to now, the longest follow-up time in the published literature is nearly 20 years, and no disease progression or recurrence has been reported in all patients (3–5, 11, 19, 21, 22). Nevertheless, previous study also highlighted that more studies are needed to further evaluate the clinical characteristics and prognosis of partial nephrectomy. In our report, two patients presented in our case report were young and middle-aged males, both of whom were found to have renal masses accidentally during physical examination, without any obvious clinical manifestations. In addition, the ultrasound results of them showed a solid cystic mass with clear borders. CT demonstrated the possibility of high-density cysts without obvious enhancement. As of yet, no disease progression or recurrence was observed in the one year follow-up. We firmly believe the morphological, immunohistochemical, and genetic information of the cases we presented would provide important material for PRNRP to become a distinct category with benign clinical outcome.

## Conclusion

PRNRP is a newly defined papillary renal cell carcinoma with characteristic pathomorphology, immunohistochemical phenotype and genetic phenotype, with promising prognosis. PRNRP should be separated from PRN and treated by partial nephrectomy instead of radical nephrectomy.

## Data availability statement

The raw data supporting the conclusions of this article will be made available by the authors, without undue reservation.

## Ethics statement

Written informed consent was obtained from the individual(s) for the publication of any potentially identifiable images or data included in this article.

## Author contributions

LZ: substantial contributions to acquisition of data and involved in drafting. ZY, YW, WX, PB, and XY: equal contributions to acquisition of data. XY: substantial contributions to data acquisition, involved in drafting. All authors contributed to the article and approved the submitted version.

## Conflict of interest

The authors declare that the research was conducted in the absence of any commercial or financial relationships that could be construed as a potential conflict of interest.

## Publisher’s note

All claims expressed in this article are solely those of the authors and do not necessarily represent those of their affiliated organizations, or those of the publisher, the editors and the reviewers. Any product that may be evaluated in this article, or claim that may be made by its manufacturer, is not guaranteed or endorsed by the publisher.
